# Incidence of hip fracture in underweight individuals: a nationwide population‐based cohort study in Korea

**DOI:** 10.1002/jcsm.13046

**Published:** 2022-07-19

**Authors:** Sangsoo Han, Jiwon Park, Hae‐Dong Jang, Sangun Nah, Joonhyeok Boo, Kyungdo Han, Jae‐Young Hong

**Affiliations:** ^1^ Department of Emergency Medicine Soonchunhyang University Bucheon Hospital Bucheon Republic of Korea; ^2^ Department of Orthopedics Korea University Hospital Ansan‐si Gyeonggi‐do Republic of Korea; ^3^ Department of Orthopaedic Surgery Soonchunhyang University Bucheon Hospital Bucheon Republic of Korea; ^4^ Department of Statistics and Actuarial Science Soongsil University Seoul Republic of Korea

**Keywords:** Cohort study, Hip fracture, Risk factor, Underweight

## Abstract

**Background:**

Hip fracture is a major public health problem worldwide and being underweight is a risk factor for fractures. Few studies have investigated the association between being underweight and hip fracture in the general population. The present study investigated the incidence of hip fracture in a large population cohort based on detailed information about the degree of underweight.

**Methods:**

A nationwide retrospective cohort study of adults ≥40 years of age included 962,533 subjects who were not overweight or obese in 2009. The incidence and risk of hip fracture occurring between 2010 and 2018 was assessed based on the degree of underweight. Based on body mass index (BMI), the study population was categorized into normal (18.50–22.99 kg/m^2^), mild (17.00–18.49 kg/m^2^), moderate (16.00–16.99 kg/m^2^), and severe underweight (<16.00 kg/m^2^) groups. Cox proportional hazards analyses were performed to calculate the hazard ratio (HR) for the hip fracture based on the degree of underweight in reference to the normal weight.

**Results:**

Compared with subjects who were normal weight, those who were classified as mild underweight (1.03/1000 person‐years (PY) increase in incidence rate (IR); adjusted HR (aHR) 1.61; 95% confidence interval (CI) 1.48–1.76), moderate underweight (2.04/1000 PY increase in IR; aHR 1.85; 95% CI 1.65–2.08), or severe underweight (4.58/1000 PY increase in IR; aHR 2.33; 95% CI 2.03–2.66) were at significantly increased risk of hip fracture.

**Conclusions:**

The severity of underweight was significantly associated with risk of hip fracture. The subdivision of underweight helps to estimate fracture risk more accurately.

## Introduction

Hip fracture is a major public health problem worldwide and has a high incidence in the elderly.^1^, ^2^ Hip fractures can lead to socio‐economic losses due to the long recovery period and can cause serious disability, reducing quality of life.^3^, ^4^ The loss of function and pain associated with hip fractures present significant challenges to patients and their families. Globally, the incidence of hip fractures and its associated burden is increasing with the aging population.^5^ Therefore, the factors associated with hip fracture have been explored in recent studies, especially modifiable risk factors affected by lifestyle such as exercise, smoking, alcohol consumption, and body mass index (BMI).^6^, ^7^

Underweight is a risk factor for cardiovascular and respiratory diseases and can increase the risk of osteoporosis and fractures.^8^, ^9^ In particular, underweight is more associated with hip fractures than with other fractures.^10^ In previous BMI and fracture studies, obesity has been specifically classified into overweight and mild/moderate/severe obesity based on BMI, and underweight (thin or slim) was mainly classified into a single group with a BMI < 18.5 kg/m^2^ due to its low prevalence (average 1–4% in developed countries).^11^, ^12^ Consequently, a unified classification of underweight can be controversial and different definitions of the degree of underweight may lead to inconsistent results. Therefore, we subdivided underweight based on the World Health Organization (WHO) classifications of mild (BMI 17.00–18.49 kg/m^2^), moderate (16.00–16.99 kg/m^2^), and severe underweight (<16.00 kg/m^2^).^13^

We hypothesized that there would be a difference in the risk of hip fracture according to the degree of underweight. The aim of this study was to investigate the relationship between hip fracture and the degree of underweight; analyses were corrected for many factors that are considered contributors to hip fracture. The study included a comprehensive population‐based dataset of adults with records on sociodemographic factors, health surveys, examinations, and medical claims from the National Health Insurance Service (NHIS) in the Republic of Korea.

## Materials and methods

### Study design and data collection

This nationwide, observational cohort study was based on claims data from the NHIS, a mandatory national health insurance system accessed by more than 97% of the population and operated by the Ministry of Health and Welfare in South Korea. The NHIS is a single insurance service managed by the Korean government: insured adults >40 years of age and workers >20 years of age undergo regular health checkups every 1–2 years. The NHIS has provided the retrospective cohort database since 2015; the database integrates the International Classification of Diseases Tenth Revision (ICD‐10), prescriptions, medical services, and costs for inpatients and outpatients. All data are anonymized, collected regularly, and carefully quality controlled.

Data for individuals who underwent health examination through the NHIS in 2009 were analysed. Subjects were excluded if they were <40 years of age or missing baseline characteristic data, had a prior diagnosis of fracture before enrolment or a fracture occurring during the 1 year lag period, or were overweight (BMI ≥ 23.0 kg/m^2^).^14^

The study protocol was approved bys the NHIS Institutional Review Board. Written informed consent from participants was waived because the NHIS data are anonymized. The Institutional Review Board also approved the study protocol (approval no. SSU‐202007‐HR‐236‐01).

### Classification of underweight

BMI was calculated by dividing body weight (kilograms) by height squared (m^2^). The study population was divided into four groups: normal weight (18.5 ≤ BMI < 23.0 kg/m^2^), mild underweight (17.5 ≤ BMI < 18.5 kg/m^2^), moderate underweight (16.5 ≤ BMI < 17.5 kg/m^2^), and severe underweight (BMI < 16.5 kg/m^2^).^13^, ^14^

### Definition of hip fracture and follow‐up

Hip fracture was defined as the ICD‐10 codes S72.0, S72.1, and S72.2 during hospitalization. Hip fracture events were identified based on NHIS medical claim records from 1 January 2010 to 31 December 2018. Participants who died during this follow‐up period were censored at the time of death.

### Demographic, social, and health‐related variables

The NHIS database yielded demographic, socio‐economic, and clinical data: sex, age, height, weight, smoking status, alcohol consumption, physical activity, household income, blood glucose, total cholesterol, blood pressure, estimated glomerular filtration rate, and any co‐morbidities. Smoking status was classified into non‐smoker, ex‐smoker, or current smoker. Alcohol consumption was classified based on the amount of alcohol consumed: non‐drinker, moderate drinker (<30 g/day), or heavy drinker (≥30 g/day).^15^, ^16^ Regular exercise was defined as at least 20 min of vigorous physical activity ≥3 days per week or ≥30 min of moderate‐intensity physical activity ≥5 days per week.^17^ Low income was defined as an income <20th percentile.

The definition of co‐morbidity based on the ICD code has been approved in previous studies.^15^, ^18^ Diabetes mellitus was defined as a fasting blood sugar level >126 mg/dL or prescription of an antidiabetic drug with ICD‐10 codes E11–E14; hypertension was defined as average blood pressure of 140/90 mmHg or more, or at least one annual prescription of an antihypertensive drug with ICD‐10 codes I10–I13 or I15; dyslipidaemia was defined as a total cholesterol level ≥240 mg/dL, or at least one annual prescription of an antihyperlipidaemic drug with ICD‐10 code E78; and chronic kidney disease (CKD) was defined as estimated glomerular filtration rate <60 mL/min/1.73 m^2^.

### Statistical analysis

Categorical variables were analysed using chi‐squared test and continuous variables were analysed using ANOVA test. The incidence rate (IR) was defined as the outcome rate per 1000 person‐years (PY), divided by the total number of hip fractures. The hazard ratios (HRs) and 95% confidence intervals (CIs) for hip fractures based on the degree of underweight were calculated based on Cox regression analysis. To investigate covariates potentially associated with hip fractures, four models were constructed. Model 1 was non‐adjusted, model 2 was adjusted for age and sex, model 3 was additionally adjusted for alcohol consumption, smoking status, regular exercise, and low income, and model 4 was fully adjusted with additional adjustments for co‐morbidities such as diabetes, hypertension, dyslipidaemia, and CKD. The cumulative incidence of hip fractures was also compared between groups using the Kaplan–Meier method. To investigate the effects of clinical conditions on the association between the degree of underweight and risk of hip fracture, the HRs for hip fractures in various subgroups were derived using Cox regression analysis as well as p‐values for interaction. Stratified subgroup analysis was conducted based on age (<65 and ≥65 years), sex, and co‐morbidities (diabetes and hypertension). SAS software (ver. 9.3; SAS Institute, Cary, NC, USA) was used for all statistical analyses. A two‐sided *P*‐value <0.05 was considered to indicate statistical significance.

## Results

**Figure 1:**
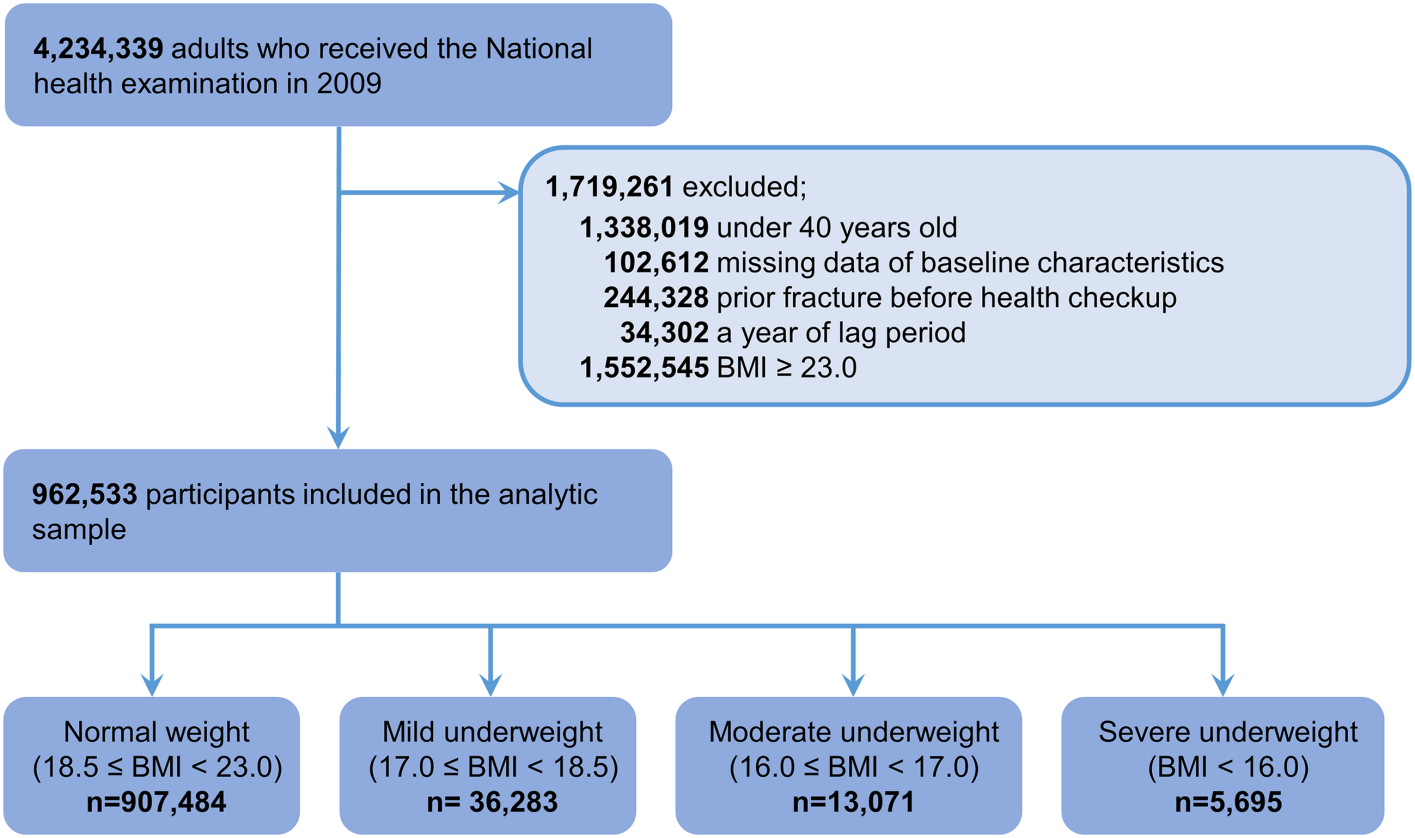
Flow chart of study population. BMI, body mass index.

In total, 4 234 339 adults underwent the national health examination in 2009. We excluded 1 719 261 individuals based on the following criteria: <40 years of age (*n* = 1 338 019), missing baseline characteristic data (*n* = 102 612), prior fracture before health checkup (*n* = 244 328), fracture occurring during the 1 year lag period (*n* = 34 302), and overweight or obese (BMI ≥ 23.0 kg/m^2^; *n* = 1 552 545). Finally, data from 962 533 individuals were included in analyses (*Figure* 1).

### Baseline characteristics

Of the 962 533 individuals included in analyses, 907 484 (94.3%) were classified as normal weight, 36 283 (3.8%) as mild underweight, 13 071 (1.4%) as moderate underweight, and 5695 (0.5%) as severe underweight. The mean BMI for each group was 21.29, 18.03, 17.06, and 15.72 kg/m^2^, respectively. Hip fracture occurred in 7717 individuals; based on the degree of underweight, these occurred in 6647 (0.73%) of those classified as normal weight, 554 (1.53%) as mild underweight, 294 (2.25%) as moderate underweight, and 222 (3.9%) as severe underweight participants. The incidence rate was highest in the severe underweight group (*P* < 0.001). Sex, age, height, weight, smoking status, alcohol consumption, regular exercise, low income, and co‐morbidities differed significantly between the four groups (*P* < 0.001). The total study population was large, so statistically significant differences were observed between groups in all variables (*Table* 1).

**Table 1:** Baseline characteristics of the study population

Variables	Normal weight	Mild underweight	Moderate underweight	Severe underweight	*P*‐value
	(18.5 ≤ BMI < 23.0)	(17.5 ≤ BMI < 18.5)	(16.5 ≤ BMI < 17.5)	(BMI < 16.5)	
	*n* = 907 484	*n* = 36 283	*n* = 13 071	*n* = 5695	
Female sex, n (%)	501 045 (55.21)	19 893 (54.83)	6678 (51.09)	3076 (54.01)	<0.001
Age, years	53.06 ± 10.45	54.36 ± 12.22	56.62 ± 13.16	60.72 ± 13.74	<0.001
≥65	759 791 (83.73)	27 818 (76.67)	9085 (69.51)	3237 (56.84)	<0.001
Height, cm	161.45 ± 8.3	161.33 ± 8.39	161.52 ± 8.62	159.93 ± 9.19	<0.001
Weight, kg	55.64 ± 6.55	47.06 ± 4.94	44.62 ± 4.77	40.35 ± 5.05	<0.001
BMI, kg/m^2^	21.29 ± 1.16	18.03 ± 0.29	17.06 ± 0.27	15.72 ± 0.8	<0.001
Smoking status, *n* (%)					<0.001
Non‐smoker	602 237 (66.36)	23 062 (63.56)	8019 (61.35)	3591 (63.06)	
Ex‐smoker	112 965 (12.45)	3593 (9.9)	1249 (9.56)	531 (9.32)	
Current smoker	192 282 (21.19)	9628 (26.54)	3803 (29.09)	1573 (27.62)	
Alcohol consumption, *n* (%)					<0.001
Non‐drinker	550 731 (60.69)	23 486 (64.73)	8698 (66.54)	4099 (71.98)	
Moderate drinker	303 503 (33.44)	10 811 (29.8)	3651 (27.93)	1283 (22.53)	
Heavy drinker	53 250 (5.87)	1986 (5.47)	722 (5.52)	313 (5.5)	
Regular exercise, *n* (%)	172 189 (18.97)	5101 (14.06)	1784 (13.65)	655 (11.5)	<0.001
Low income, *n* (%)	167 385 (18.44)	7076 (19.5)	2557 (19.56)	1174 (20.61)	<0.001
Co‐morbidities, *n* (%)					
Diabetes	71 043 (7.83)	2238 (6.17)	925 (7.08)	500 (8.78)	<0.001
Hypertension	216 730 (23.88)	6781 (18.69)	2685 (20.54)	1423 (24.99)	<0.001
Dyslipidaemia	146 740 (16.17)	3663 (10.1)	1288 (9.85)	609 (10.69)	<0.001
CKD	63 163 (6.96)	2498 (6.88)	960 (7.34)	571 (10.03)	<0.001
Hip fracture, *n* (%)	6647 (0.73)	554 (1.53)	294 (2.25)	222 (3.9)	<0.001

BMI, body mass index; CKD, chronic kidney disease.

### Association between hip fracture and the degree of underweight

**Figure 2:**
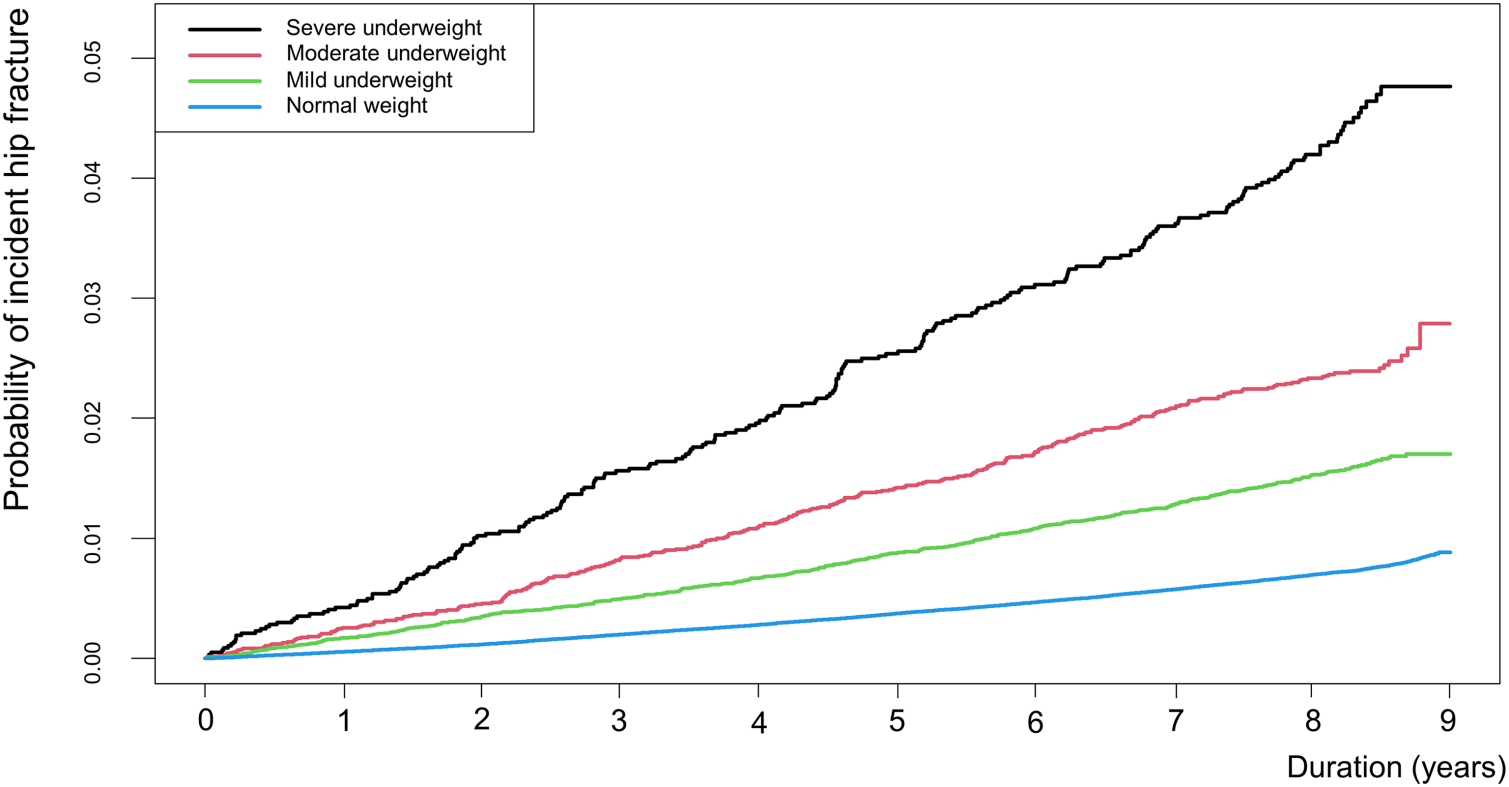
Kaplan–Meier estimates for the probability of incident hip fracture up to 9 years based on underweight categories.

Cox proportional hazards regression analyses were performed to investigate the relationship between the degree of underweight and hip fracture. After multivariable adjustment (Model 4; demographics, socioeconomic factors, lifestyle behaviours, and co‐morbidities), individuals classified as mild underweight (1.03/1000 PY increase in IR; HR 1.61; 95% CI 1.48–1.76), moderate underweight (2.04/1000 PY increase in IR; HR 1.85; 95% CI 1.65–2.08), and severe underweight (4.58/1000 PY increase in IR; HR 2.33; 95% CI 2.03–2.66) had a significantly increased risk of hip fracture events compared with those of normal weight (*Table* 2). *Figure* 2 presents the Kaplan–Meier estimate curves between groups. The severe underweight group had a significantly higher incidence of hip fracture at all time points (log‐rank test, *P* < 0.001).

**Table 2:** Association between degree of underweight and risk of hip fracture

Group	Events	PY	IR (per 1000 PY)	HR (95% CI)			
				Model 1	Model 2	Model 3	Model 4
Normal weight	6647	7 398 522.5	0.90	1 (Reference)	1 (Reference)	1 (Reference)	1 (Reference)
Mild underweight	554	286 671.7	1.93	2.17 (1.99–2.36)	1.57 (1.44–1.71)	1.52 (1.39–1.65)	1.61 (1.48–1.76)
Moderate underweight	294	100 147.7	2.94	3.31 (2.94–3.72)	1.80 (1.61–2.03)	1.74 (1.54–1.95)	1.85 (1.65–2.08)
Severe underweight	222	40 521.8	5.48	6.24 (5.46–7.14)	2.27 (1.99–2.60)	2.16 (1.89–2.47)	2.33 (2.03–2.66)

HR, hazard ratio; IR, incidence rate; CI, confidence interval; PY, person‐years; CKD, chronic kidney disease.

Model 1: Non‐adjusted; Model 2: Adjusted for age and sex; Model 3: Adjusted for age, sex, smoking status, alcohol consumption, low income, and regular exercise; Model 4: Adjusted for age, sex, smoking status, alcohol consumption, low income, regular exercise, diabetes, hypertension, dyslipidaemia, and CKD.

### Subgroup analysis

Subgroup analyses were conducted by stratifying the study population based on age, sex, smoking status, alcohol consumption, household income, and co‐morbidities. *Table* 3 lists the estimated HRs based on subgroup using multivariable Cox proportional hazards regression. Individuals classified as severe underweight and ≥65 years of age, male, and without diabetes or hypertension were at a significantly increased risk of hip fracture compared with normal weight participants. No significant differences were observed in other subgroup analyses, which included smoking status, alcohol consumption, household income, dyslipidaemia, and CKD (*P* > 0.05).

**Table 3:** Stratified analyses based on age, sex, diabetes, and hypertension

Stratified variables	Category	HR (95% CI)			*P* for interaction
		Mild underweight	Moderate underweight	Severe underweight	
Age (years)	≥65	2.01 (1.68–2.40)	2.70 (2.09–3.48)	4.37 (3.18–6.00)	<0.001
	<65	1.53 (1.39–1.69)	1.74 (4.52–1.98)	2.16 (1.86–2.50)	
Sex	Male	1.88 (1.67–2.12)	2.20 (1.87–2.58)	3.32 (2.73–4.03)	<0.001
	Female	1.39 (1.22–1.57)	1.57 (1.33‐–1.87)	1.84 (1.52–2.21)	
Diabetes	No	1.57 (1.42–1.73)	1.82 (1.60–2.07)	2.42 (2.10–2.80)	0.012
	Yes	1.85 (1.51–2.27)	2.02 (1.52–2.69)	1.80 (1.22–2.65)	
Hypertension	No	1.51 (1.33–1.70)	2.06 (1.76–2.40)	1.51 (1.33–1.70)	<0.001
	Yes	1.74 (1.53–1.97)	1.59 (1.33–1.92)	1.86 (1.50–2.31)	

Comparison with the normal weight group as the reference group. Adjusted for age, sex, smoking status, alcohol consumption, low income, regular exercise, diabetes, hypertension, dyslipidaemia, and chronic kidney disease (CKD).

HR, hazard ratio; CI, confidence interval.

## Discussion

This large, population‐based cohort study using the NHIS database in the Republic of Korea yielded real‐world evidence that risk of hip fracture increased with increased severity of underweight compared with normal weight. In particular, individuals classified as severe underweight (BMI < 16 kg/m^2^) had a significantly higher risk of hip fracture events. These findings help to more accurately estimate the risk of hip fractures through weight subdivision. Clinicians should educate patients to gain weight through nutritional management and weight training to reduce the risk of hip fractures in underweight patients.

Demographic factors such as age and sex are well‐known risk factors for hip fracture. The risk of hip fracture increases with age and females have a higher risk of hip fractures than males due to lower bone density.^19^ Lifestyle factors such as smoking, alcohol consumption, and regular exercise are also reportedly associated with hip fracture.^20^, ^21^ Chronic diseases such as diabetes and hypertension can also affect hip fractures.^22^, ^23^ We used the data available in the NHIS database as much as possible to adjust mediators and confounders that may affect hip fracture occurrence.

We found that the more severe the degree of underweight, the higher the risk of hip fracture. This result can be explained by the following hypothesis. First, underweight is associated with sarcopenia, and muscle can protect bone from severe trauma and stress.^24^ Therefore, a decrease in muscle mass and function is associated with an increase in fall‐related fractures such as hip fracture.^25^, ^26^ Second, with increasing underweight, the thickness of the soft tissue surrounding the greater trochanter decreases; soft tissue can reduce impact loads through decreases in effective stiffness and/or increases in energy absorption.^27^, ^28^ Third, underweight can be associated with deficiencies in nutrients, such as calcium, vitamin D, and protein, which can lead to reduced bone density and bone metabolism.^29^ Calcium deficiency can decrease the bone mineral density (BMD), and low vitamin D levels has been shown to be associated with defective mineralization of collagenous matrix (osteoid).^30^, ^31^ Also, protein depletion may affect the bone remodelling process by reducing the production of insulin‐like growth factor 1.^32^ Finally, the more severe underweight, the lower the BMD, and the BMD of the femoral neck is significantly lower in severe underweight subjects than in normal weight subjects.^29^

We found that more severe underweight was associated with a greater accretion in the incidence of hip fractures in elderly individuals >65 years of age compared with younger individuals. Fall‐related injuries are usually more likely to occur in the elderly due to the presence of individual conditions such as muscle weakness and impaired vision and balance.^33^ Although we expected worse outcomes in females than in males due to conditions such as osteoporosis,^34^ the results showed that males classified as severe underweight were at greater risk of hip fracture than females. This result is in agreement with a previous study in which the BMD of the femoral neck was measured in two groups of underweight and normal weight subjects, and the difference in BMD between the two groups was greater in men than in women.^29^ Therefore, BMD may be further decreased as the degree of underweight becomes more severe in males; however, additional research is needed to confirm this hypothesis.

The risk of sarcopenia is increased in diabetic subjects compared with healthy individuals.^35^ Additionally, in patients with diabetes, BMD may be reduced by hyperglycaemia, oxidative stress, and the accumulation of advanced glycation end products that compromise collagen properties, increase marrow adiposity, release inflammatory factors and adipokines from visceral fat, and potentially alter the function of osteocytes.^36^ In the present study, in the moderate underweight group, the risk of hip fracture was higher in the diabetic group than in the non‐diabetic group. However, the opposite result was observed in the severe underweight group; more research is needed to explore these findings. In the present study, in the severe underweight group, individuals with hypertension had a higher risk of hip fracture. Hypertension is associated with abnormalities in calcium metabolism, such as increased urinary calcium excretion, and persistent hypercalciuria in hypertensive patients may increase the risk of BMD loss.^37^ A previous meta‐analysis reported that hypertension can significantly reduce BMD in the human body including the femoral neck (95% CI: −0.09 to −0.02).^38^

The present study had several notable strengths. To the best of our knowledge, it is the first to investigate the association between degree of underweight and hip fracture using nationwide data. Additionally, data from the NHIS are representative of the country and the sample size was large, so the results of this study can be generalized to the Korean population. We also performed extensive stratified analyses using other covariates including age, sex, and co‐morbidities, which enhanced the reliability of the results.

However, this study also had several limitations. First, the study design was retrospective. We tried to adjust for as many confounders as possible, but other confounders may exist. Also, because we used the NHIS database, individuals who did not receive medical services were likely excluded, so the incidence of hip fracture may have been underestimated. Second, the NHIS database does not include data regarding patient BMD. Third, although Korea is rapidly becoming Westernized, our findings should be generalized with caution; ethnicity and geography may affect the occurrence of hip fracture. Previous studies exploring racial differences and hip fractures have reported that individuals of Polynesian descent, including Pasifika people, tend to have a lower risk of hip fractures than European individuals due to high BMD.^19^, ^39^ In addition, we classified normal weights as 18.5 ≤ BMI < 23.0 kg/m^2^ according to the WHO classification for the Asia‐Pacific region.^14^ Fourth, we did not consider several co‐morbidities such as hyperthyroidism and malassimilation syndrome which are well‐known risk factors for both underweight and osteoporosis.^40^, ^41^ Also, we did not consider laboratory parameters such as calcium, protein, and vitamin D; these are potential confounders that are related with the fracture.^30^, ^31^, ^32^ Further well‐designed studies are needed to overcome these limitations.

## Conclusion

When underweight was further subdivided based on the degree of severity, a proportionally increased risk of hip fractures was observed. These findings add to clinical evidence that the subdivision of the underweight can help to more accurately assess hip fracture risk.

## Conflict of interest

Authors have no conflict of interest.

## Funding

The authors received no financial support for the research.
